# Macronutrient intake and alertness during night shifts – the time interval matters

**DOI:** 10.3389/fnut.2023.1245420

**Published:** 2023-11-09

**Authors:** Mariëlle G. de Rijk, Alexander P. J. van Eekelen, Sanne Boesveldt, Elly Kaldenberg, Tineke Holwerda, Ceciel J. M. Lansink, Edith J. M. Feskens, Jeanne H. M. de Vries

**Affiliations:** ^1^Division of Human Nutrition and Health, Wageningen University and Research, Wageningen, Netherlands; ^2^Circadian NL, Amsterdam, Netherlands; ^3^Sanavis, Spijk, Netherlands; ^4^Hospital Gelderse Vallei, Ede, Netherlands; ^5^Deventer Hospital, Deventer, Netherlands

**Keywords:** shift work, diet, nutrients, sustained attention, occupational health, fatigue, timing

## Abstract

**Background:**

Working night shifts is associated with higher safety risks due to shift work-related fatigue. Nutrition, especially certain (macro) nutrient compositions, has been suggested to reduce fatigue, however, results of studies are contradictory. This could be explained by differences in the time interval investigated between the consumption of a meal and measurement of cognitive performance. Therefore, this observational study investigated the association between macronutrient intake and objective alertness at different time intervals during the night shift in nurses.

**Methods:**

128 nurses, aged 20–61 years, completed an alertness test (Psychomotor Vigilance Task) during the night shift and a 24-h dietary recall after the night shift. This was repeated three times, always on the first night shift in a night shift series. The associations between macronutrient intake 0 to 1, 1 to 2, and 2 to 3 h before the PVT with alertness during the night shift were analyzed through Linear Mixed Models. The basic model was adjusted for age and gender and the adjusted model additionally for BMI, start time of PVT and energy and caffeine intake during the relevant time interval.

**Results:**

Protein intake was not associated with objective alertness levels, while fat and carbohydrates intake had opposite associations with objective alertness levels over similar time intervals. Fat intake up to 1 h prior to the PVT was borderline associated with a longer median reaction time (RT) (ß = 9.00 ms/10 g fat, 95% CI: −0.21, 18.20), while a higher carbohydrate intake up to 1 h prior to the PVT was borderline associated with shorter median RTs (ß = −3.89, 95% CI: −7.85, 0.06). A higher fat intake 2 to 3 h prior to the PVT was associated with less lapses (log transformed ß = −0.16; 95% CI: −0.31, −0.02), while a higher carbohydrate intake 2 to 3 h prior to the PVT was associated with more lapses (ß = 0.06, 95% CI: 0.01, 0.12).

**Conclusion:**

Our results contribute to understanding the association between macronutrient intake, as part of a mixed meal, and alertness levels. Conflicting results from previous studies may probably be due to time differences between macronutrient intake and alertness testing.

## 1. Introduction

In healthcare, continuous care is provided 24 h a day, 7 days a week. This means that health care workers, including nurses, have to work outside the conventional working hours, including at night (1–3). Unfortunately, night shift work is associated with higher health and safety risks. For example, night shift workers have a 30 percent increased risk of making (medical) errors or having accidents than day shift workers (4–6). This is mainly caused by shift work-related fatigue (5).

Several studies have investigated how shift-work related fatigue can be reduced, e.g., by using lamps that simulate daylight during the night shift and implementing behaviour strategies (7). In addition to sleep and exercise strategies, nutrition strategies can be applied to reduce fatigue and thereby improve alertness during the night (8, 9). For example, consumption of meals during the night shift with specific (macro) nutrient compositions may reduce fatigue and improve cognitive performance (8, 10).

Studies investigating the effect of (macro) nutrient composition on cognitive performance (e.g., sustained attention, memory, processing speed) are limited and relatively old. Most of these studies have primarily focused on glucose intake and were conducted during the daytime (10, 11). Compared to sweeteners, glucose has been shown to improve memory, but might also benefit other cognitive functions (10). However, it is not yet clear whether cognitive performance is solely affected by glucose intake or by consumption of carbohydrate-rich foods in general (11), or what the impact of other macronutrients (protein and fat) would be. One study showed that carbohydrate-rich meals increase sleepiness, but only in obese night shift workers (12). Another study showed that a protein-rich breakfast, as well as a protein and carbohydrate-balanced breakfast, resulted in better overall cognitive performance directly after the meal than a carbohydrate-rich breakfast. In addition, a carbohydrate-rich breakfast resulted in better peripheral attention but worse central attention after 135 min than a protein and carbohydrate-balanced breakfast (13). Yet another pilot study found that cognitive performance during the night improved after a test meal that was higher in fat and lower in carbohydrates than the baseline meal that was relatively low in fat and high in carbohydrates (8). Hence, the effects of different macronutrient composition of meals are not always very distinct and sometimes even contradictory (9, 10, 12, 14).

A factor that could contribute to these contradictory results is the time interval between the consumption of a meal and measurement of cognitive performance. As mentioned earlier, Fischer et al. (13), reported differences in cognitive performance tested directly and 135 min after the meal. Besides meal related aspects, the various types of tests that have been used to measure cognitive performance may also contribute to the contradictory study outcomes (10, 14). Most studies focusing on the effect of glucose intake found that it was positively associated with the domains of short term and delayed memory performance, while the effect of a specific macronutrient composition of a meal was suggested to be better reflected in tasks that involve sustained attention (10). In addition to this objective measure of alertness, it is recommended to assess subjective alertness (14, 15). While the correlation between subjective and objective alertness tends to be modest, particularly during the biological night (15), subjective alertness may be seen as a perceivable benefit that consequently enhances the motivation to comply with dietary intervention strategies.

Although the recent advancements in the studies of diet and cognitive performance are promising, a better understanding is needed of the association between diet and sustained attention especially in night shift workers (9, 10). Since a meal does not solely consist of a single macronutrient, research should particularly focus on the differential effects of the specific types of macronutrients in a mixed meal, taking into account the time interval between the meal and the cognitive task (14). Therefore, we investigated the association between macronutrient intake with objective and subjective alertness, measured during different time periods in nurses during the night shift. The results of this study can be used to develop nutrition intervention strategies as a vital part of occupational health and safety management programs in shift work.

## 2. Methods

### 2.1. Participants

Nurses were recruited in three hospitals located in the surrounding area of Wageningen, Netherlands. They were invited via email by the researcher and via advertisements on the internal website for employees of the hospitals. Nurses were included in the study if they were working the night shift for at least 6 months, were not using drugs that could cause or reduce sleep problems, were not using daylight lamps on the workplace during the night shift, were eating according to a Dutch eating pattern (2 bread meals and 1 hot meal) and were not donating blood during the data collection period as this may cause fatigue (16). In total, we included 164 nurses in the study, 24 of whom dropped out before nutritional data was collected. We additionally excluded nine smokers and three nurses with missing data for smoking, as smokers typically go outside for their cigarette break, which could have affected the results of the alertness test. The final study sample consisted of 128 nurses. All nurses gave written consent before the start of the study.

### 2.2. Study design and procedure

This observational study was conducted between April 2015 and July 2018. At the start of the data collection period, nurses’ height and body weight were measured and they filled out a demographic questionnaire. Thereafter, based on the night shift timetables, nurses were scheduled for the measurements. They were asked to complete an alertness test once during the night shift and to complete a 24-h dietary recall after the night shift. These measurements were repeated three times, always during the first night of three different night shift series with at least one month in between. The study was approved by the Medical Ethical Committee of Wageningen University and Research (ABR: NL54414.081.15) and was conducted according to the principles of the Declaration of Helsinki.

### 2.3. Objective and subjective alertness

Objective alertness was assessed by the Psycho Vigilance Test (PVT). The PVT is a validated 10-min visual reaction time task that measures sustained attention (17). This task was carried out on a computer between 2:00 AM and 5:00 AM, around the circadian nadir for alertness. Nurses were instructed to press the space bar as quickly as possible when a white circle, the stimulus, appeared on the black screen. The visual stimulus appeared every 2 to 10 s for 10 min at a fixed point on the screen. For each completed PVT, we determined the mean reaction time (RT; ms), mean reciprocal response time (1/RT), number of lapses (RT > 500 ms).

Subjective alertness was assessed by the 7-point Samn Perelli Scale (SPS). At the start of the PVT, nurses were asked to complete the SPS. With the SPS, nursers are asked how they feel at that moment. The answer scores ranges from 1 (“fully alert, wide awake”) to 7 (“completely exhausted, unable to function effectively”) (18).

### 2.4. Dietary intake

Each 24-h dietary recall was completed by the nurses on the day after the first night shift. Nurses were asked to record all the foods and drinks they consumed during 24 h; from the evening meal prior to the first night shift until the evening meal after the night shift. They were also asked to write down the time of day when each food or drink was consumed. Each recall was self-administered via Compl-eat, a web-based program, based on a validated technique to increase the accuracy of dietary recalls (19), and includes foods used in a Dutch food pattern. Portion sizes of foods or recipes were reported by household measures, standard portion sizes, or weights in gram. When the reported recall was not clear (e.g., missing portion sizes), the participant was called or emailed by the researcher or research dietitian for clarification. Average daily intakes of energy, macronutrient, alcohol and caffeine of each participant were calculated by multiplying frequency of consumption of food items by portion size and energy and nutrient content using the 2013 Dutch Food Composition Table (20). In addition, based on the reported start and end times of the night shift, it was determined what each participant consumed during the night shift, and what was consumed 0 to 1 h, 1 to 2 h and 2 to 3 h before the PVT.

### 2.5. Anthropometrics and demographics

Height and body weight were measured without shoes by trained researchers and used to calculate body mass index (BMI) in kg/m^2^. Height was determined using a stadiometer to the nearest 0.5 cm. Body weight was assessed using an analogue scale to the nearest 0.5 kg or digital weighing scale to the nearest 0.1 kg depending on the hospital. The demographic questionnaire included questions about, e.g., education, smoking, and working hours.

### 2.6. Statistical analysis

Data was analyzed using IBM SPSS Statistics 25. *p*-values below 0.05 were considered statistically significant. Raw data were checked for quality (outliers, omissions) and normality. If normally distributed, data are presented in mean ± SD or in *n* (%), otherwise in median and interquartile range. Three cases of outliers (average median RT > 600 ms) in alertness levels were detected, of which two in the same participant. To meet the assumption for linear mixed models (LMM) that the residuals were normally distributed, this participant was excluded from the data analysis. Therefore, the final study sample includes 127 participants. Eight of these participants completed only one 24-h dietary recall, 15 participants two recalls, 101 participants three recalls, and three participants four recalls, resulting in a total of 353 recalls.

A paired *t*-test was performed to investigate differences between the macronutrient intake in energy percentages during the whole day (including the night shift) and solely during the night shift. We calculated Spearman correlation coefficients between subjective alertness and median RT, 1/RT and log transformed number of lapses. To analyze the associations between macronutrient intake 0 to 1 h, 1 to 2 h and 2 to 3 h before the PVT with alertness during the night shift, we used LMM with alertness as a dependent variable (objective and subjective measures separately) and each macronutrient intake in gram as the independent variables. We also controlled for dependence amongst the repeated measurements in each nurse and controlled for cluster effects within the three hospitals. In the basic model, we adjusted for age and gender and in the adjusted model we additionally adjusted for BMI, start time of PVT, and energy and caffeine intake during the relevant time interval.

#### 2.6.1. Misreporting of daily energy intake

Misreporting of daily energy intake is a widely known phenomenon (21). Potential recalls of poor validity were identified by using the principles of the Goldberg cut-off evaluating the ratio energy intake: basal metabolic rate (EI: BMR) at the individual level (21, 22). BMR was estimated by the Oxford equation from body weight, taking into account age, and gender (23). Recalls with EI: BMR values lower than 0.87 and higher than 2.75 were classified as misreported recalls, which was the case for 22.7% of the recalls. All statistical analyses were performed with and without misreported recalls. However, because excluding misreported recalls did not alter the associations, we presented only the results of the total study sample.

## 3. Results

### 3.1. Participant characteristics

The majority of the nurses were female (92.1%) and were classified as having normal BMI (55.9%) (Table 1). Nurses started their night shift between 10:00 and 11:15 PM and ended their night shift between 7:00 and 8:15 AM. Eighty-one percent of the nurses (*n* = 103) commuted sometimes or always by car. Of these nurses, 26 nurses reported to have ever fallen asleep behind the wheel after a night shift, and 5 nurses had actually been involved in an accident after a night shift because of fatigue.

**Table 1:** Anthropometrics and demographics of all nurses (*n* = 127) and by sex in mean ± SD, median (IQR) or *n* (%).

	Overall		Women		Men	
	*n* = 127		*n* = 117		*n* = 10	
Age, years (IQR)	42.3	(29.4–51.3)	43.4	(30.0–51.4)	33.3	(23.4–48.8)
Weight, kg ± SD	72.6	13.0	71.7	12.8	82.8	10.9
Height, cm ± SD	170.4	7.3	169.2	5.8	184.1	9.3
*Body Mass Index, kg/m^2^ ± SD*	25.0	4.1	25.0	4.1	24.6	24.6
Underweight (<18 kg/m^2^), *n* (%)	1	(0.8)	1	(0.9)	0	(0.0)
Normal (18–25 kg/m^2^), *n* (%)	71	(55.9)	65	(55.6)	6	(60.0)
Overweight (25–30 kg/m^2^), *n* (%)	45	(35.4)	42	(35.9)	3	(30.0)
Obese (>30 kg/m^2^), *n* (%)	10	(7.9)	9	(7.7)	1	(10.0)
Education						
Intermediate education, *n* (%)	65	(51.2)	61	(52.1)	4	(40.0)
Higher education, *n* (%)	60	(47.2)	54	(46.2)	6	(60.0)
Married/cohabiting						
Yes, *n* (%)	104	(81.9)	97	(82.9)	7	(70.0)
Children						
Yes, *n* (%)	69	(54.3)	64	(54.7)	5	(50)
Hours of employment, per week ±SD	28.9	5.7	28.5	5.7	34.4	2.1
Night shifts series/month, series (IQR)	2.0	(1.0–4.0)	2.0	(1.0–4.0)	2.5	(1.0–4.0)
Night shifts/serie, nights ±SD	2.7	1.0	2.6	1.0	3.6	1.1
Night shift experience, years ±SD	17.0	11.6	17.3	11.5	13.2	12.7
Commute by car, *n* (%)	103	(81.1)	97	(82.9)	6	(60.0)
Fallen asleep behind the wheel, *n*	26		24		2	
Involved in an accident caused by fatigue, *n*	5.0		5		0	

### 3.2. Objective and subjective alertness

The PVT, including the SPS question, was on average done at 3:08 ± 0:34 AM. Nurses had a median reaction time (RT) of 412.9 [IQR: 382.2–461.6] ms and 9.7 [IQR: 4.0–19.3] lapses on the PVT. They scored on average 3.2 ± 0.7 out of a score of 7 on the SPS, which is considered as “Okay, somewhat fresh” (18). The SPS score was positively correlated with the number of total lapses (log transformed) (*r* = 0.162, *p* = 0.002) and median RT (*r* = 0.117, *p* = 0.028) and inversely correlated with 1/RT (*r* = −0.129, *p* = 0.015). The objective and subjective alertness levels did not significantly differ between the three study periods.

### 3.3. Dietary intake

All nurses consumed foods or drinks during the night shift, with a median of 3.3 (IQR:2.3–4) eating occasions at varying time points. Nurses consumed foods or drinks in 49.3% of the recalls 1 h before the PVT, in 44.8% of the recalls 1 to 2 h before the PVT and in 33.7% of the recalls 2 to 3 h before the PVT. During the night shift, dietary intake consisted on average ± SD of 15.6 ± 4.5 energy percent (en%) protein, 29.2 ± 10.1 en% fat, 52.3 ± 11.1 en% carbohydrates and 25.4 ± 12.2 en% mono- and disaccharides (Table 2). These relative intakes of macronutrients significantly differed (*p* < 0.001) from those consumed during the whole day, especially for carbohydrates and mono- and disaccharides that were higher during the night shift.

**Table 2:** Average dietary intake of nurses (*n* = 127) derived from 24-h recalls presented in median (IQR) intake and mean ± SD energy percentage.

	Whole day				During night shift				0 to 1 h before PVT		1 to 2 h before PVT		2 to 3 h before PVT	
	Mean	SD	En%	SD	Mean	SD	En%	SD	Median	(IQR)	Median	(IQR)	Median	(IQR)
Energy, kcal	1,697	504			618	230			78	(0.0–210.6)	52	(0.0–161.4)	7	(0.0–74.3)
Energy, kJ	7,118	2,113			2,597	964			331	(0.0–883.6)	220	(0.0–681.7)	29	(0.0–313.9)
*Protein, g*	70.3	21.2	16.9	3.7	23.6	9.5	15.6	4.5	2.3	(0.0–8.4)	1.2	(0.0–6.1)	0.2	(0.0–2.3)
Plant protein, g	28.4	9.5	6.7	1.5	11.3	5.1	7.3	2.0	1.1	(0.0–3.6)	0.5	(0.0–3.0)	0.1	(0.0–1.2)
Animal protein, g	42.0	17.2	10.2	4.0	12.3	6.8	8.3	4.5	0.5	(0.0–4.1)	0.1	(0.0–2.9)	0.0	(0.0–0.7)
*Fat, g*	63.1	25.9	32.7	7.5	20.8	11.6	29.2	10.1	1.6	(0.0–7.1)	0.8	(0.0–5.2)	0.1	(0.0–2.4)
Saturated fatty acids, g	24.5	10.9	12.7	3.7	8.7	5.6	12.3	5.4	0.6	(0.0–2.8)	0.3	(0.0–1.7)	0.0	(0.0–0.8)
MUFA, g	20.8	9.6	0.4	0.2	6.3	4.2	0.4	0.3	0.4	(0.0–1.8)	0.2	(0.0–1.2)	0.0	(0.0–0.7)
PUFA, g	11.8	5.6	10.8	3.1	3.7	2.6	8.8	4.3	0.2	(0.0–1.1)	0.1	(0.0–0.8)	0.0	(0.0–0.3)
Trans fatty acids, g	0.8	0.5	6.1	2.0	0.3	0.3	5.2	2.7	0.0	(0.0–0.1)	0.0	(0.0–0.0)	0.0	(0.0–0.0)
Carbohydrates, g	197.9	64.1	47.0	7.8	80.0	33.3	52.3	11.1	10.9	(0.0–24.5)	7.7	(0.0–18.5)	0.4	(0.0–10.3)
Mono-disaccharides, g	86.8	38.6	20.6	6.9	37.9	22.6	25.4	12.2	3.9	(0.0–9.9)	1.9	(0.0–8.5)	0.2	(0.0.0–4)
Polysaccharides, g	111.0	37.3	26.4	5.4	42.0	21.4	26.9	9.6	3.8	(0.0–13.5)	1.7	(0.0–10.0)	0.0	(0.0–4.8)
*Dietary fibers, g*	21.2	6.9	2.6	0.7	8.4	3.4	2.8	1.0	1.0	(0.0–2.4)	0.5	(0.0–2.3)	0.0	(0.0–0.8)
Alcohol, g	0.7	2.5	0.3	1.2	0.0	0.0	0.0	0.0	0.0	(0.0–0.0)	0.0	(0.0–0.0)	0.0	(0.0–0.0)
Water, g	2,332	756			1,007	409			164	(50–263)	126	(0.0–246.3)	54	(0.0–156.6)
Caffeine, mg	108.4	118.2			55.9	63.3			0.0	(0.0–13.8)	0.0	(0.0–2.1)	0.0	(0.0–0.0)

IQR, interquartile range; MUFA, mono unsaturated fatty acids; PUFA, poly unsaturated fatty acids.

### 3.4. Macronutrient intake and alertness

The associations of macronutrient intake for different time intervals to the PVT and objective and subjective measures of alertness are presented in Table 3; Supplementary Table S1. Significant associations were observed with objective but not with subjective alertness levels.

**Table 3:** Association between (specific) macronutrient intakes 0 to 1, 1 to 2 and 2 to 3 h prior to PVT and objective and subjective alertness in nurses (*n* = 127) during the night shift.

	Median reaction time						Reciprocal reaction time					
	Basic model*			Adjusted model^†^			Basic model*			Adjusted model^†^		
	**β**	95% CI	*p* value	**β**	95% CI	*p* value	**β**	95% CI	*p* value	**β**	95% CI	*p* value
**Protein/10 g**												
< 1 h before PVT	3.74	(−0.53, 8.01)	0.085	2.17	(−8.89, 13.23)	0.700	-0.03	(−0.05, 0.00)	0.056	-0.01	(−0.08, 0.05)	0.676
1–2 h before PVT	−6.14	(−10.52, −1.76)	0.006	−6.42	(−18.03, 5.19)	0.277	0.04	(0.02, 0.07)	0.002	0.00	(−0.07, 0.07)	0.987
2–3 h before PVT	5.14	(−2.66, 12.93)	0.195	−1.71	(−17.26, 13.84)	0.829	−0.02	(−0.07, 0.02)	0.351	0.00	(−0.10, 0.09)	0.982
**Fat/10 g**												
< 1 h before PVT	4.48	(0.30, 8.67)	0.036	9.00	(−0.21, 18.20)	0.055	−0.03	(−0.05, 0.00)	0.026	−0.05	(−0.11, 0.00)	0.074
1–2 h before PVT	−5.25	(−9.54, −0.97)	0.016	−2.43	(−12.9, 8.04)	0.648	0.04	(0.02, 0.07)	0.001	0.03	(−0.03, 0.09)	0.348
2–3 h before PVT	1.76	(−4.92, 8.43)	0.605	−15.31	(−31.16, 0.54)	0.058	0.00	(−0.04, 0.04)	0.922	0.08	(−0.02, 0.18)	0.098
**Carbohydrates/10 g**												
< 1 h before PVT	0.74	(−0.84, 2.32)	0.358	−3.89	(−7.85, 0.06)	0.054	−0.01	(−0.02, 0.00)	0.266	0.02	(0.00, 0.05)	0.064
1–2 h before PVT	−1.87	(−3.60, −0.15)	0.034	1.88	(−2.79, 6.54)	0.429	0.02	(0.01, 0.03)	0.004	−0.01	(−0.04, 0.02)	0.412
2–3 h before PVT	2.67	(0.36, 4.98)	0.024	6.04	(−0.35, 12.43)	0.064	−0.01	(−0.03, 0.00)	0.095	−0.03	(−0.07, 0.01)	0.129
**Mono-disaccharides/10 g**												
< 1 h before PVT	−0.74	(−3.73, 2.25)	0.627	−3.83	(−7.53, −0.13)	0.042	0.00	(−0.01, 0.02)	0.724	0.02	(0.00, 0.05)	0.043
1–2 h before PVT	−1.52	(−5.17, 2.12)	0.411	1.80	(−2.44, 6.04)	0.405	0.02	(0.00, 0.04)	0.099	−0.01	(−0.03, 0.02)	0.652
2–3 h before PVT	4.32	(0.39, 8.25)	0.031	4.08	(−1.88, 10.04)	0.179	−0.02	(−0.04, 0.00)	0.117	−0.02	(−0.05, 0.02)	0.318
**Polysaccharides/10 g**												
< 1 h before PVT	2.15	(−0.21, 4.51)	0.074	0.83	(−4.12, 5.78)	0.742	−0.01	(−0.03, 0.00)	0.048	−0.01	(−0.04, 0.02)	0.681
1–2 h before PVT	−3.06	(−5.47, −0.66)	0.013	−0.36	(−5.89, 5.17)	0.898	0.02	(0.01, 0.04)	0.002	−0.01	(−0.04, 0.03)	0.669
2–3 h before PVT	4.08	(−0.24, 8.39)	0.064	3.19	(−6.67, 13.04)	0.525	−0.02	(−0.04, 0.01)	0.169	−0.02	(−0.08, 0.04)	0.504
**Dietary fibres/10 g**												
< 1 h before PVT	9.29	(−3.87, 22.45)	0.165	−10.97	(−36.83, 14.9)	0.405	−0.07	(−0.15, 0.01)	0.077	0.03	(−0.12, 0.19)	0.662
1–2 h before PVT	−11.04	(−24.78, 2.69)	0.114	14.72	(−11.59, 41.04)	0.271	0.09	(0.01, 0.18)	0.028	−0.11	(−0.26, 0.05)	0.174
2–3 h before PVT	14.56	(−8.65, 37.76)	0.218	−13.53	(−53.85, 26.79)	0.509	−0.07	(−0.21, 0.08)	0.367	0.06	(−0.19, 0.30)	0.658

	Number of lapses (log)						Samn perelli scale					
	Basic model*			Adjusted model^†^			Basic model*			Adjusted model^†^		
	**β**	95% CI	*p* value	**β**	95% CI	*p* value	**β**	95% CI	*p* value	**β**	95% CI	*p* value
**Protein/10 g**												
< 1 h before PVT	0.02	(−0.02, 0.06)	0.257	−0.02	(−0.12, 0.09)	0.753	−0.01	(−0.11, 0.10)	0.878	−0.04	(−0.31, 0.23)	0.778
1–2 h before PVT	−0.02	(−0.06, 0.02)	0.303	0.01	(−0.10, 0.12)	0.816	0.01	(−0.10, 0.13)	0.807	−0.08	(−0.39, 0.23)	0.600
2–3 h before PVT	0.01	(−0.06, 0.08)	0.753	−0.03	(−0.17, 0.11)	0.697	−0.01	(−0.21, 0.18)	0.900	−0.26	(−0.66, 0.14)	0.201
**Fat/10 g**												
< 1 h before PVT	0.03	(−0.01, 0.07)	0.151	0.05	(−0.04, 0.13)	0.271	−0.01	(−0.12, 0.09)	0.794	−0.02	(−0.26, 0.23)	0.887
1–2 h before PVT	−0.02	(−0.06, 0.01)	0.212	−0.03	(−0.12, 0.07)	0.561	0.06	(−0.05, 0.17)	0.293	0.20	(−0.08, 0.47)	0.166
2–3 h before PVT	−0.02	(−0.08, 0.04)	0.607	−0.16	(−0.31, −0.02)	0.029	0.03	(−0.13, 0.18)	0.746	−0.03	(−0.44, 0.38)	0.884
**Carbohydrates/10 g**												
< 1 h before PVT	0.01	(−0.01, 0.02)	0.317	−0.02	(−0.05, 0.02)	0.358	0.00	(−0.04, 0.04)	0.905	0.01	(−0.09, 0.12)	0.847
1–2 h before PVT	−0.01	(−0.02, 0.01)	0.300	0.01	(−0.03, 0.05)	0.659	0.00	(−0.04, 0.05)	0.967	−0.07	(−0.19, 0.06)	0.292
2–3 h before PVT	0.02	(0.00, 0.04)	0.119	0.06	(0.01, 0.12)	0.031	0.03	(−0.03, 0.08)	0.350	0.06	(−0.11, 0.23)	0.491
**Mono-disaccharides/10 g**												
< 1 h before PVT	0.00	(−0.03, 0.03)	0.930	−0.02	(−0.06, 0.01)	0.197	0.01	(−0.06, 0.09)	0.769	0.02	(−0.08, 0.12)	0.688
1–2 h before PVT	−0.01	(−0.05, 0.02)	0.497	0.00	(−0.04, 0.04)	0.939	0.00	(−0.10, 0.09)	0.917	−0.03	(−0.14, 0.08)	0.593
2–3 h before PVT	0.02	(−0.01, 0.06)	0.214	0.02	(−0.03, 0.07)	0.457	0.05	(−0.05, 0.14)	0.333	0.05	(−0.11, 0.20)	0.552
**Polysaccharides/10 g**												
< 1 h before PVT	0.02	(0.00, 0.04)	0.118	0.01	(−0.03, 0.06)	0.565	0.00	(−0.06, 0.06)	0.963	−0.02	(−0.14, 0.11)	0.778
1–2 h before PVT	−0.01	(−0.03, 0.01)	0.314	0.01	(−0.04, 0.06)	0.651	0.00	(−0.06, 0.07)	0.890	−0.04	(−0.19, 0.11)	0.601
2–3 h before PVT	0.03	(−0.01, 0.07)	0.125	0.09	(0.00, 0.18)	0.043	0.04	(−0.07, 0.14)	0.495	0.02	(−0.23, 0.26)	0.901
**Dietary fibres/10 g**												
< 1 h before PVT	0.08	(−0.04, 0.20)	0.183	−0.05	(−0.28, 0.18)	0.656	0.08	(−0.25, 0.41)	0.631	0.21	(−0.45, 0.88)	0.522
1–2 h before PVT	−0.04	(−0.16, 0.09)	0.536	0.08	(−0.16, 0.32)	0.501	−0.05	(−0.41, 0.30)	0.775	−0.66	(−1.38, 0.05)	0.068
2–3 h before PVT	0.08	(−0.13, 0.29)	0.432	0.00	(−0.37, 0.36)	0.994	0.10	(−0.48, 0.67)	0.739	−0.35	(−1.32, 0.61)	0.473

^*^Basic models are adjusted for age and gender. † Models are additional adjusted for BMI, start time PVT, and energy and caffeine intake of the relevant time interval.

#### 3.4.1. Protein

Irrespective of age and gender, absolute protein intake was inversely associated with median RT and positively associated with 1/RT, but only 1 to 2 h before the PVT. However, this was not reflected by significantly less lapses. Moreover, these associations disappeared after additional adjustments for BMI, start time of PVT and energy and caffeine intake during the relevant time interval.

#### 3.4.2. Fat

A higher fat intake up to 1 h prior to the PVT was associated with a longer median RT (ß = 4.48 ms/10 g fat, 95% CI: 0.30, 8.67) and a lower 1/RT (ß = −0.03 ms/10 g fat, 95% CI: −0.05, 0.00). This association was slightly attenuated after the additional adjustments.

No associations were found between fat intake 1 to 2 h prior to the PVT and objective alertness levels.

A higher fat intake 2 to 3 h prior to the PVT was associated with slightly shorter median RTs (ß = −15.31 ms/10 g fat, 95% CI: −31.16, 0.54, *p* = 0.058). This was also reflected by less lapses; every 10 gram of fat consumed 2 to 3 h before the PVT was associated with 0.16 less lapses (log transformed; 95% CI: 0.02, 0.31, *p* = 0.029).

#### 3.4.3. Carbohydrates

In contrast to a higher fat intake, a higher absolute carbohydrate intake up to 1 h prior to the PVT was associated with a slightly shorter median RT (ß = −3.89, 95% CI: −7.85, 0.06, *p* = 0.054) and 1/RT (ß = 0.02, 95% CI: 0.00, 0.05, *p* = 0.064) in the adjusted model. Especially a higher mono- and disaccharides consumption was associated with a shorter median RT (ß = −3.83, 95% CI: −7.53, −0.13, *p* = 0.042) and better 1/RT (ß = 0.02, 95% CI: 0.00, 0.05, *p* = 0.043) in the adjusted model.

Just as for fat, no associations were found between carbohydrate intake 1 to 2 h prior to the PVT and objective alertness levels.

Also in contrast with fat intake, a higher carbohydrate consumption 2 to 3 h prior to the PVT was associated with longer median RTs (ß = 2.67, 95% CI: 0.36, 4.98, *p* = 0.024) in the basic model and borderline associated with longer median RTs (ß = 6.04, 95% CI: −0.35, 12.43, *p* = 0.064) after additional adjustments. A higher carbohydrate consumption and a higher polysaccharide consumption 2 to 3 h before the PVT was also associated with more lapses (ß = 0.06, 95% CI: 0.01, 0.12, *p* = 0.031 and ß = 0.09, 95% CI: 0.00, 0.18, *p* = 0.043, respectively, in the adjusted model).

### 3.5. Covariates and alertness

We also examined the association between alertness levels and covariates separately (Table 4). Age was inversely associated with SPS (ß = −0.01, *p* = 0.019), meaning that an older age was associated with feeling more alert. Gender and BMI were not associated with objective or subjective alertness. The start time of the PVT was significantly associated with both objective and subjective alertness levels, indicating that nurses were less alert later during the night shift (*p* < 0.001). Energy intake was associated with better median RT (ß = −4.01, *p* < 0.001) and 1/RT (ß = 0.03, *p* < 0.001) and less lapses (ß = −0.02, *p* = 0.015) after adjustment for the other covariates, but only when energy was consumed 1 to 2 h before the PVT and not at the other time points. Caffeine intake up to 1 h before starting the PVT was associated with worse median RT (ß = 1.38, *p* = 0.047) and 1/RT (ß = −0.01, *p* = 0.036). This means that a higher caffeine intake was associated with a slower reaction time. However, this was not reflected by more lapses or higher feeling of sleepiness (higher SPS score).

**Table 4:** Association between covariates and objective and subjective alertness in nurses (*n* = 127) during the night shift.

	Median RT			Reciprocal RT			Number of lapses (Log)			SPS		
	**β**	95% CI	*p* value	**β**	95% CI	*p* value	**β**	95% CI	*p* value	**β**	95% CI	*p* value
Age, years*	0.19	(−0.54, 0.92)	0.607	−0.00	(−0.01, 0.00)	0.853	−0.00	(−0.01, 0.00)	0.588	−0.01	(−0.02, 0.00)	0.019
Gender, male*	−11.54	(−42.73, 19.65)	0.465	0.08	(−0.12, 0.28)	0.410	−0.21	(−0.47, 0.04)	0.099	0.01	(−0.46, 0.47)	0.977
BMI, kg/m2*	0.37	(−1.68, 2.43)	0.721	0.00	(−0.02, 0.01)	0.594	0.00	(−0.02, 0.02)	0.882	−0.01	(−0.04, 0.02)	0.368
Start time PVT, min*	0.16	(0.08, 0.24)	<0.001	−0.00	(−0.00, −0.00)	<0.001	0.00	(0.00, 0.00)	<0.001	0.00	(0.00, 0.01)	<0.001
Energy/100 kcal^†^												
< 1 h before PVT	1.25	(−0.66, 3.16)	0.199	−0.01	(−0.02, 0.00)	0.137	0.01	(−0.01, 0.03)	0.173	0.00	(−0.05, 0.05)	0.879
1–2 h before PVT	−4.01	(−5.98, −2.05)	<0.001	0.03	(0.02, 0.04)	<0.001	−0.02	(−0.04, 0.00)	0.015	−0.02	(−0.07, 0.04)	0.564
2–3 h before PVT	1.13	(−1.86, 4.11)	0.457	−0.00	(−0.02, 0.02)	0.735	0.00	(−0.02, 0.03)	0.859	0.00	(−0.07, 0.07)	0.971
Caffeine/10 mg^†^												
< 1 h before PVT	1.38	(0.02, 2.74)	0.047	−0.01	(−0.02, 0.00)	0.036	0.00	(−0.01, 0.02)	0.526	0.01	(−0.03, 0.04)	0.631
1–2 h before PVT	1.23	(−0.20, 2.66)	0.091	−0.01	(−0.02, 0.00)	0.059	0.01	(0.00, 0.02)	0.206	0.03	(−0.01, 0.06)	0.143
2–3 h before PVT	1.17	(−0.14, 2.48)	0.080	−0.01	(−0.01, 0.00)	0.165	0.00	(−0.01, 0.01)	0.782	0.00	(−0.03, 0.03)	0.930

^*^Models are adjusted for age and gender. † Models are additional adjusted for BMI, start time PVT and energy or caffeine intake of the relevant time interval. All models are controlled for dependence amongst the repeated measurements for each nurse and for cluster effects within the three hospitals.

## 4. Discussion

This study is one of the first that reports on the association between macronutrient composition as part of mixed meals, meal timing, and (sustained) alertness in a real life night shift setting. There are two key findings. First, we found opposite associations for objective alertness levels for the intake of fat and carbohydrates. These associations were not only opposite between fat and carbohydrates, but also between fat and carbohydrate intake up to 1 h, and 2 to 3 h before the PVT. Fat consumption 1 h prior to the PVT was associated with poorer alertness levels, while fat consumption 2 to 3 h prior to the PVT was associated with better alertness levels, and for carbohydrates we observed opposite time dependent associations. Second, we found no associations between the intake of any of the macronutrients (protein, fat and carbohydrates) 1 to 2 h before the PVT and objective alertness levels. This suggests a kind of tipping point with fat and carbohydrate intake 1 to 2 h before the PVT and their association with alertness. However, an overall higher energy intake 1 to 2 h before the PVT was associated with better alertness levels. We did not find an association between protein intake and alertness levels, nor did we observe a difference in timing. No associations were found between macronutrient intake and subjective levels of alertness.

The opposite time dependent associations for fat and carbohydrate intake and objective alertness levels could explain why some studies showed positive effects of macronutrient intake on alertness while others showed negative effects. Of the previous studies on this topic, only two relatively old (pilot) studies were conducted during the night shift (8, 24). One study did measure cognitive performance at several time points during the night but averaged the scores, by which time differences could have been missed (8) and the other did not provide information about the time at which cognitive performance was measured (24). Moreover, neither of the studies presented details about consumption time and macronutrient composition. Therefore, it is hard to conclude from these earlier studies how macronutrient intake is associated with objective alertness levels and how the time interval between meal and alertness testing affected their results, especially during the night.

Studies that were conducted during the day around breakfast and lunchtime do support the time dependent associations of fat and carbohydrate intake with objective alertness levels. Our finding of a time dependent association between carbohydrate intake and objective alertness is in line with the findings from a review of Dye et al. (14). They also suggest that carbohydrate intake in general seems to deteriorate attention and reaction time but that it depends on the type of carbohydrates, time of the day and ratio of carbohydrates to protein (14). For example, glucose intake was associated with improved short-term and delayed memory immediately after a meal but was also associated with impaired memory after 60 min (11, 14). Indeed, we also observed a positive association with objective alertness and the intake of mono- and disaccharides 1 h before the PVT but not 2 to 3 h before the PVT. Moreover, absolute intake of carbohydrates 2 to 3 h before the PVT was associated with borderline significant slower reaction times and significantly more lapses. This association could be driven by the polysaccharide intake as a higher polysaccharide intake was also associated with more lapses. Lloyd et al. (25) showed that a high-carbohydrate low-fat meal resulted in slower reaction times 90 and 150 min after ingestion compared to a medium-carbohydrate medium-fat meal. This also supports the idea that a higher carbohydrate consumption has a negative impact on alertness levels after about two hours, as found in our current study. On the other hand it could also be that higher fat intake resulted in better reaction times. Fischer et al. (26) showed that overall cognitive performance was best after a pure fat meal, compared to meals that consisted of solely protein or carbohydrates. However, this finding was not replicated in a study of Jones et al. (11), and -again- timing could be a reason for these inconclusive results. Jones et al. (11) tested cognitive performance 15 min and 60 min after the test meal, whereas Fischer et al. tested cognitive performance 60, 120 and 180 min after the test meal. Although Jones et al. observed some beneficial effects of fat intake compared to placebo on the cognitive domain processing 15 min after the test meal, this beneficial effect was not maintained after 60 min, nor was it more beneficial compared to the protein or carbohydrate intake. They suggested that they possibly missed the time frame to detect beneficial effects of fat intake (11).

Another point of attention is the difficulty and by this the domain of cognitive performance that was tested; each domain can be more or less sensitive to manipulations of the intake of one specific macronutrient or macronutrient composition in general (10, 14). The effect of macronutrients, and especially glucose, are highest for memory processes (10, 14). The exact mechanisms behind it have not been elucidated, but it has been shown that a rise in glucose levels – glucose is the primary source of energy for the brain – is associated with better performance on memory and reaction time tasks (10). Also, tasks that require a greater or longer cognitive demand are possibly more sensitive to macronutrient manipulations (10). We here assessed the cognitive domain sustained attention, which requires a longer cognitive demand and is therefore suggested to be more affected by macronutrient manipulations compared to most other cognitive domains (10).

Taking the above into account we carefully conclude that time is an important factor when measuring associations between fat and carbohydrate intake and objective alertness levels. Carbohydrates, especially the mono and disaccharides, are associated with better objective alertness levels directly after a meal but are associated with lower levels of objective alertness after two hours, probably due to the presence of polysaccharides. The association between fat and objective alertness is less clear cut, especially directly after the meal. Two hours after intake, fat may be associated with better objective alertness levels, but this warrants further investigations.

In contrast to previous literature, we did not find an association between protein intake and alertness levels, nor did we observe a difference in timing. Even though there was an inverse association between protein intake 1 to 2 h before the PVT and objective alertness levels after adjustment for age and gender, this association disappeared after additional adjustment for BMI, start time of PVT, and energy and caffeine intake. Some studies found a positive effect of protein intake on cognitive performance. These studies focused more on single macronutrients in a meal or at protein rich meals relatively to carbohydrate rich meals (11, 13, 26), whereas we examined absolute protein intake as part of a mixed meal. The enhancing effect of protein may be less pronounced in a mixed meal or absolute protein intake was too low to find an association with alertness. Furthermore, protein intake may still be associated with better cognitive performance in comparison to carbohydrate intake, but less pronounced as fat intake. Fischer et al. (13), for example, suggested that a balanced or protein rich-meal will result in better overall performance than a medium or high carbohydrate-rich meal. This may be due to less variation in blood glucose levels, as high variation in the glucose levels could impair cognitive performance (13). Another explanation could be that protein intake has the least negative effects on alertness levels compared to fat and carbohydrate intake.

Similar to protein intake, we did not find associations between fat and carbohydrate intake 1 to 2 h before the PVT and objective alertness level. On the other hand, a higher (overall) energy intake 1 to 2 h prior to the PVT was associated with better objective alertness levels. This may suggest that, to improve alertness 1 to 2 h after consumption, it is not so much the macronutrient composition of the meal but rather the consumption of food as such which is most relevant. A positive association between energy intake, independent of the macronutrient composition, and cognitive performance is also suggested by the review of Dye (14). However, the association between energy intake and cognitive performance was not linear. Large meals in terms of energy intake may deteriorate alertness levels even further during the night shift, while smaller meals may not (8, 27). In our study energy intake during the different time frames was only small and might thus explain why we found a positive association with alertness levels.

We did not find associations of macronutrient intake and subjective alertness levels. Other studies are also inconclusive about these associations. In the study of Paz and Berry (24) participants’ regular diet seemed to result in better subjective alertness levels than a high protein and high carbohydrate diet, although this was not statistically significant. In the study of Nehme et al. (12) carbohydrates seemed to result in lower subjective alertness levels, but only in overweight participants. Similar to objective alertness levels, subjective alertness levels could be associated with energy intake independently. Landström et al. (28) found a direct positive effect of consuming food on subjective feelings of alertness in sleep deprived persons. However, these effects disappeared after half an hour (28), a time frame that we did not assess and which could explain why we did not see these effects. Moreover, all the meals provided in the study of Landström et al. contained carbohydrates to a greater or lesser extent (28). Altogether, it remains uncertain whether there is an association between macronutrient intake and subjective alertness levels. Moreover, our results indicated a negligible correlation between subjective and objective alertness (29). This weak correlation is not unexpected, as subjective alertness might be overestimated especially during the night (15). Therefore, better and more sensitive methods are needed to assess subjective alertness, specifically over a longer time period.

In our models, caffeine intake was associated with median reaction time (RT) and reciprocal reaction time (1/RT), but not the way we expected. More caffeine was associated with a higher median RT and lower 1/RT, and thus with poorer objective alertness levels. In addition, the number of lapses and subjective feeling of alertness were not associated with caffeine intake. Explanations could be that the intake of caffeine was too low to improve alertness or that individuals that were the most tired had the most caffeine (i.e., reverse causation).

We observed that the time at which the PVT and SPS was performed was associated with both subjective and objective alertness. The PVT was performed between 2:00 AM and 5:00 AM and nurses were more tired later in the night shift. Grant et al. (30) also found a significant effect of time of day on number of lapses and median RT, where the highest number of lapses and median RTs were seen at 04:00 AM. This effect of time on PVT performance was also seen in other studies with shorter versions of the PVT (31, 32). Ganesan et al. (31) found an increase in number of lapses and RT over time during the night shift measured by a 5-min PVT. Gupta et al. (32) also found an increase in number of lapses and RT at 03:00 AM compared to 8:30 PM measured by a 3-min PVT in shift workers. This deterioration in alertness, independent of food intake, is not only observed during the night but also around lunch time (14, 33). It cannot be prevented, but food intake can strengthen or mitigate it. Thus, besides the time interval between consumption and testing, it is also important at what time of day alertness levels are measured.

This study is to our knowledge the first study that investigated the association between macronutrient composition together with meal timing, and (sustained) alertness in nurses in a real life night shift setting. Despite that under-reporting was present in the current study, the nurses were very motivated to take part in this study and were well instructed to successfully complete the 24-h recalls. Also, removal of identified poor reports of energy intake in some nurses did not alter the associations that we found. Therefore, we had reliable information not only about what they ate, but also about when and how often they ate before, during and after a night shift, and how this was associated with alertness (34). Another strength of this study is our relatively large sample size compared to other studies. This allowed us to investigate specific time frames and to deal with the large variation in dietary intake. Lastly, this study showed the value that research in this field can have for night shift workers. Besides the increased risks of (medical) errors or having work-related accidents during the night shifts compared to day shifts (5), this study showed that nurses also reported fatigue related road traffic accidents after their night shift, which is in line with earlier findings (35, 36). These results supports the importance of proper nutrition strategies for night shift workers to promote safety during and after the night shift.

The observational design of this real life study comes with some limitations. One limitation related to this observational design is that we cannot draw conclusions about causal relationships between macronutrient intake and alertness levels. Moreover, we have to interpret the observed associations with caution, as the reported regression coefficients are rather small and we performed multiple comparisons. In addition, some participants consumed foods up to 1 h as well as 2 to 3 h before the PVT. We did not adjust for food consumption up to 1 h before the PVT, which means this could have influenced the associations between macronutrient intake 2 to 3 h before the PVT and alertness. Another limitation is the uncontrolled environment in which nurses performed the PVT and where nurses could be distracted by colleagues or patients. However, they could restart the PVT when necessary, and therefore we assume this has had little impact on the results. Moreover, other confounders that we did not collect such as menstrual cycle, physical activity during the night shift, and napping, sleep duration, and sleep quality prior to the night shift could have influenced alertness levels and could have resulted in more power when taken into account. Although we observed that half of the nurses took a nap before the night shift, we do not know when and how long they napped. It is presumed that in a more controlled setting the results could lead to distinct associations and more clinical relevant results.

Lastly, the study sample consisted almost exclusively of female nurses. Results remained the same when men were excluded (Supplementary Table S1), but the number of men was too small to study them separately. Therefore, it remains speculative whether these associations also holds true in a (larger) male sample. Moreover, participating nurses might have been more interested in the role of nutrition in health, well-being and safety than other nurses or other night shift professionals, and could therefore be health oriented. In a more unhealthy study sample, there may be more variation in macronutrient intake and this could potentially result in more distinct associations with alertness. The type of work might have also affected alertness levels; work that is more monotonous and less distracting can lead to lower alertness levels and therefore be more susceptible to changes in macronutrient composition. Therefore, the results of this study cannot be generalized to other night shift working professions. It would be interesting to see how macronutrient composition is associated with alertness levels in other night shift working professions, especially in those who have an unhealthier lifestyle and relative higher BMI, such as production employees. It is suggested that a higher BMI could mediate the effect of carbohydrates on sleepiness (12).

In conclusion, our results contribute to understanding the association between macronutrient intake, as part of a mixed meal, and alertness levels. It seems plausible that conflicting results from previous studies are due to the time between macronutrient intake and alertness testing. Fat intake was associated with lower alertness levels shortly after consumption, but could be associated with better alertness levels 2 to 3 h after intake. The opposite seems plausible for carbohydrate intake. Protein consumption did not appear to have a distinct association with alertness levels or appeared to have the least negative impact on alertness levels compared to fat and carbohydrate consumption. Based on these results we would advise a small macronutrient-balanced meal or a high-protein meal during the night shift to maintain and achieve the most optimal alertness levels. However, it is not yet known which macronutrient composition would be optimal. This should be tested in an intervention study, in which meal timing, meal frequency, and energy intake are included.

The practical implications of this observational study suggest that individuals, when eating during the night, should prioritize low-fat and low-carbohydrate food choices. Additionally, careful consideration should be given to the timing of meals. Consuming foods 1 to 2 hours before tasks requiring optimal alertness levels seems to have the least detrimental effect. In light of this, a recommendation could be made to schedule meals approximately 1 to 2 h before the circadian nadir and 1 to 2 h before returning home, ensuring optimal alertness and well-being.

## Data availability statement

The raw data supporting the conclusions of this article will be made available by the authors, without undue reservation.

## Ethics statement

The studies involving humans were approved by Medical Ethical Committee of Wageningen University and Research. The studies were conducted in accordance with the local legislation and institutional requirements. The participants provided their written informed consent to participate in this study.

## Author contributions

MR, JV, AE, and EK designed the study (project conception, development of overall study plan). MR conducted the research with help from TH and CL. MR did the data analysis, with counselling of EF. MR, JV, SB, and EF wrote the manuscript. All authors contributed to the article and approved the submitted version.
