# Supplementary material for: Macronutrient intake and alertness during night shifts – the time interval matters
Source: Front Nutr. 2023 Nov 9;10:1245420. doi: 10.3389/fnut.2023.1245420 (PMC10665514; doi:10.3389/fnut.2023.1245420)
Supplement: Supplementary file 1 [file Table_1.DOCX]

Supplemental table 1. Association between (specific) macronutrient intakes 0 to 1, 1 to 2 and 2 to 3 hours prior to PVT and objective and subjective alertness in *female* nurses (n=117) during the night shift.

|  | **Median Reaction Time*** | | | **Reciprocal Reaction Time*** | | | **Number of Lapses (log)*** | | | **Samn Perelli Scale*** | | |
| --- | --- | --- | --- | --- | --- | --- | --- | --- | --- | --- | --- | --- |
|  | **ß** | **95% CI** | **P value** | **ß** | **95% CI** | **P value** | **ß** | **95% CI** | **P value** | **ß** | **95% CI** | **P value** |
| **Protein / 10g** |  |  |  |  |  |  |  |  |  |  |  |  |
| < 1 hour before PVT | 4.18 | (-7.51, 15.88) | 0.482 | -0.02 | (-0.09, 0.05) | 0.514 | 0.00 | (-0.11, 0.10) | 0.983 | 0.01 | (-0.27, 0.30) | 0.929 |
| 1-2 hours before PVT | -7.76 | (-19.91, 4.40) | 0.210 | 0.01 | (-0.06, 0.08) | 0.760 | 0.00 | (-0.11, 0.11) | 0.982 | -0.06 | (-0.38, 0.26) | 0.704 |
| 2-3 hours before PVT | -1.82 | (-17.58, 13.94) | 0.820 | 0.00 | (-0.09, 0.09) | 0.997 | -0.03 | (-0.17, 0.11) | 0.692 | -0.28 | (-0.69, 0.13) | 0.180 |
| **Fat / 10g** |  |  |  |  |  |  |  |  |  |  |  |  |
| < 1 hour before PVT | 9.06 | (-0.36, 18.48) | 0.059 | -0.05 | (-0.11, 0.01) | 0.080 | 0.04 | (-0.04, 0.13) | 0.344 | -0.02 | (-0.27, 0.24) | 0.888 |
| 1-2 hours before PVT | -1.56 | (-13.45, 10.34) | 0.797 | 0.02 | (-0.05, 0.09) | 0.551 | -0.01 | (-0.11, 0.10) | 0.916 | 0.21 | (-0.10, 0.53) | 0.174 |
| 2-3 hours before PVT | -15.75 | (-32.21, 0.71) | 0.061 | 0.08 | (-0.02, 0.18) | 0.105 | -0.17 | (-0.32, -0.02) | 0.022 | -0.08 | (-0.51, 0.35) | 0.708 |
| **Carbohydrates / 10g** |  |  |  |  |  |  |  |  |  |  |  |  |
| < 1 hour before PVT | -4.20 | (-8.25, -0.15) | 0.042 | 0.02 | (0.00, 0.05) | 0.056 | -0.02 | (-0.05, 0.02) | 0.372 | 0.00 | (-0.11, 0.11) | 0.969 |
| 1-2 hours before PVT | 1.83 | (-3.37, 7.03) | 0.488 | -0.01 | (-0.04, 0.02) | 0.540 | 0.00 | (-0.05, 0.05) | 0.939 | -0.07 | (-0.21, 0.06) | 0.308 |
| 2-3 hours before PVT | 6.27 | (-0.37, 12.91) | 0.064 | -0.03 | (-0.07, 0.01) | 0.131 | 0.07 | (0.01, 0.13) | 0.024 | 0.08 | (-0.09, 0.26) | 0.358 |
| **Mono-disaccharides / 10g** |  |  |  |  |  |  |  |  |  |  |  |  |
| < 1 hour before PVT | -3.32 | (-7.20, 0.55) | 0.092 | 0.02 | (0.00, 0.04) | 0.077 | -0.02 | (-0.05, 0.02) | 0.273 | 0.03 | (-0.08, 0.13) | 0.623 |
| 1-2 hours before PVT | 2.15 | (-2.95, 7.25) | 0.407 | 0.00 | (-0.03, 0.03) | 0.912 | -0.01 | (-0.06, 0.03) | 0.590 | -0.03 | (-0.16, 0.10) | 0.668 |
| 2-3 hours before PVT | 3.84 | (-2.26, 9.95) | 0.216 | -0.02 | (-0.05, 0.02) | 0.367 | 0.02 | (-0.03, 0.08) | 0.446 | 0.06 | (-0.10, 0.22) | 0.464 |
| **Polysaccharides / 10g** |  |  |  |  |  |  |  |  |  |  |  |  |
| < 1 hour before PVT | -0.81 | (-6.18, 4.56) | 0.767 | 0.00 | (-0.03, 0.03) | 0.979 | 0.01 | (-0.04, 0.06) | 0.723 | -0.04 | (-0.18, 0.09) | 0.547 |
| 1-2 hours before PVT | -0.51 | (-6.31, 5.28) | 0.862 | -0.01 | (-0.04, 0.02) | 0.561 | 0.02 | (-0.03, 0.07) | 0.480 | -0.05 | (-0.20, 0.11) | 0.532 |
| 2-3 hours before PVT | 3.81 | (-6.15, 13.77) | 0.452 | -0.02 | (-0.08, 0.04) | 0.440 | 0.09 | (0.01, 0.18) | 0.038 | 0.03 | (-0.22, 0.28) | 0.807 |
| **Dietary fibres / 10g** |  |  |  |  |  |  |  |  |  |  |  |  |
| < 1 hour before PVT | -8.37 | (-35.48, 18.75) | 0.544 | 0.03 | (-0.13, 0.19) | 0.748 | -0.04 | (-0.28, 0.20) | 0.769 | 0.30 | (-0.40, 1.00) | 0.396 |
| 1-2 hours before PVT | 13.41 | (-14.68, 41.5) | 0.348 | -0.10 | (-0.26, 0.07) | 0.251 | 0.05 | (-0.20, 0.31) | 0.674 | -0.60 | (-1.36, 0.15) | 0.116 |
| 2-3 hours before PVT | -13.45 | (-55.03, 28.12) | 0.524 | 0.06 | (-0.19, 0.31) | 0.657 | 0.00 | (-0.37, 0.37) | 0.995 | -0.29 | (-1.29, 0.70) | 0.562 |

* Adjusted models: adjusted for age, BMI, start time PVT, and energy and caffeine intake of the relevant time interval.
